# Hospital Nursing Staff Perceptions of Resources Provided by Their Organizations During the COVID-19 Pandemic

**DOI:** 10.1177/2165079920987543

**Published:** 2021-01-29

**Authors:** Hyeonmi Cho, Knar Sagherian, Linsey M. Steege

**Affiliations:** 1 University of Wisconsin–Madison; 2 The University of Tennessee, Knoxville

**Keywords:** nursing staff, hospital, resource, support, COVID-19

## Abstract

**Background:**

The coronavirus disease 2019 (COVID-19) pandemic has profoundly impacted the health and psychological well-being of hospital nursing staff. While additional support is needed to better cope with increased job stressors, little is known about what types of hospital resources have been provided and how nursing staff perceive them. This study addressed this gap by describing nursing staff perceptions of resources provided by hospitals during the COVID-19 pandemic in the United States.

**Methods:**

Registered nurses and nursing assistants who were working in hospitals during the pandemic were recruited to an online survey via social media posts and emails between May and June 2020. A total of 360 free-text responses to an open-ended survey question were analyzed using content analysis.

**Results:**

Over half of participants reported being provided with hospital resources. “Basic needs” resources that included food on-site, groceries, and childcare support were the most frequently reported compared with four other types of resources (personal health and safe practice, financial support, managerial support, communication). Four themes emerged related to staff perceptions of support: community support, unequal benefits, decreasing resources, and insufficient personal protective equipment.

**Conclusion:**

Our findings can assist organizational leaders in the planning and allocation of different types of resources that are meaningful to nursing staff and thus ensure sustainability, optimal performance, and worker well-being during crises.

The rapid spread of the novel coronavirus disease 2019 (COVID-19) has tremendously impacted the health of the global community. As of October 2020, 35.5 million individuals have been infected and 1,042,000 individuals have died from COVID-19 complications worldwide (World Health Organization, 2020). This pandemic has created serious psychological health problems in the general population and in the health care workforce (Giusti et al., 2020).

In hospitals, nursing staff were on the frontline providing care in close physical proximity to patients with COVID-19 and had direct risk of being exposed to the virus (Sim, 2020). Nurses worked long hours under extremely dynamic conditions, had heavy workloads, and carried the fear of becoming infected and transmitting the virus to family and friends (Liu et al., 2020; Schroeder et al., 2020). Many have experienced a decline in psychological well-being, such as increased anxiety, depression, and post-traumatic stress (Giusti et al., 2020), that may interfere with the safe delivery of high-quality care. Moreover, personal stressors during the pandemic such as availability of childcare services, school closures, financial problems, and dramatic changes in work–family balance may have further exacerbated levels of anxiety and distress among nursing staff who remained on the job (Shanafelt et al., 2020; Sinclair et al., 2020).

Health care organizations play a critical role during crises as they can procure essential resources, promote a psychosocial safety climate, and mitigate the negative effects of the job (Loh et al., 2018; Ripp et al., 2020). Ripp and colleagues (2020) reported on the New York City Mount Sinai Health System’s organized efforts to promote and maintain the emotional well-being of health care workers during the pandemic. The taskforce that consisted of behavioral health, human resources, and well-being leaders identified three priority areas to address for their health care workers: (a) meet basic daily needs such as food, housing, and childcare; (b) improve communications for dissemination of reliable and timely information; and (c) develop psychosocial and mental health resources (Ripp et al., 2020).

Organizational resources, when readily available, help workers cope with job and life stressors and maintain healthy work environments (Kelloway & Day, 2005). Motivation and commitment are attained when health care leaders recognize and value, and better understand the unmet needs of employees during high job demands (Benson & Dundis, 2003). Previous studies have shown that health care workers were more willing to work during crises when organizational resources such as monetary incentives, childcare, and personal protective equipment (PPE) were available (Damery et al., 2009; Garrett et al., 2009). Availability of organizational resources to nursing staff can help ensure personal safety and health that will translate to safe performance and delivery of quality care.

During the COVID-19 pandemic, nursing staff in general have reported resource challenges such as availability of PPE and food access among others (Liu et al., 2020; Ripp et al., 2020). Little is known about what types of organizational resources were provided to hospital nursing staff from their own perspectives in the United States. It is important to address this gap and identify unmet needs and opportunities for hospital leadership to plan for and allocate different types of resources during COVID-19 and for future public health emergencies. Our study aimed to describe nursing staff perceptions of organizational resources provided by hospitals during the pandemic. We explored how many nursing staff reported receiving additional resources, what types of resources were provided, and how nursing staff perceived these organizational resources.

## Methods

### Design

This study used cross-sectional data collected as a part of a larger parent study that examined the psychological health and well-being and experiences of hospital nursing staff during the COVID-19 pandemic in the United States (Sagherian et al., 2020). A free-response item was included in the survey that asked participants the following question: “During the COVID-19 pandemic, did your hospital provide you with any extra resources?” All responses to this item were used in this study. Institutional review board approvals were obtained from the University of Tennessee, Knoxville and the University of Wisconsin–Madison. Participants were given detailed information about the study purpose and any potential risks at the start of the survey.

### Sample and Data Collection

Convenience sampling was used to recruit registered nurses (RNs) and certified nursing assistants who were providing direct patient care during the COVID-19 pandemic in hospitals in the United States. Participants were invited to an online survey via social media posts and emails between May and June 2020. Nursing staff who were on vacation or maternity leave and had leadership roles were not part of the study. Among 587 nursing staff who participated in the parent study, 360 participants completed the free-response item.

### Data Analysis

Summative content analysis was used to analyze the responses. This analytic approach is commonly used for free-response survey questions to identify certain words and quantify them to understand a situation (Hsieh & Shannon, 2005). Two coders independently reviewed all the responses and identified categorical codes for whether resources were provided, resource types and other themes related to nursing staff perceptions of resources. The two coders then discussed the identified categorical codes and agreed upon a final list of codes. One coder coded each response using the defined categorical codes and then the other coder verified all the codes. Any disagreement between the coders was resolved through discussion. Following the coding of all responses, a frequency analysis was conducted to identify the most frequently cited resource types.

## Results

Most participants who completed the free-response item were RNs (*n* = 332, 92.2%), female (*n* = 343, 95.3%), White (*n* = 317, 88.3%), married/living with a partner (*n* = 245, 68.1%), and were between 20 and 67 years of age (*M* = 38.00, *SD* = 11.08). Almost half of the participants had dependent children (*n* = 165, 46%) and 17.6% (*n* = 63) had dependent elderly in their homes.

More than one third of participants reported having 3 to 8 years of nursing experience (*n* = 148, 41.3%), were on night shifts (*n* = 124, 34.4%), and worked on average more than 40 hours per week (*n* = 122, 34.3%) during the COVID-19 pandemic. Most participants worked extended shifts (≥10 hours) (*n* = 303, 86.1%) and reported caring for patients with COVID-19 (*n* = 241, 67.7%).

Responses to the item “During the COVID-19 pandemic, did your hospital provide you with any extra resources?” were somewhat mixed across the sample. Of the 360 participants, 211 (58.6%) reported that hospitals provided them with at least one extra resource and 149 (41.4%) reported that they did not receive anything additional during the COVID-19 pandemic. Of the 211 participants who reported their hospitals providing at least one resource, many listed multiple resources that were each coded separately (see Table 1). For example, one participant responded, “Mental health resources, food during work hours, childcare.” In contrast, a number of participants who reported not being provided with resources were emphatic in their negative responses. For example, one participant responded, “None, no extra resources at all. No freebies, no help of any kind.” Others reported that some support was provided; however, there were cuts (e.g., work hours, pay) and lack of sufficient resources (PPE). For example, one participant responded, “Some [resources provided]; but they also laid us off by 40% pay reduction.” The findings of the free-response question are organized as follows: (a) types of resources nursing staff reported receiving from hospitals and (b) descriptions of four additional emerging themes related to nursing staff’s perceptions of resources.

**Table 1.** Types of Resources Nursing Staff Reported Receiving From Their Hospitals During COVID-19

Types of resources offered	Frequency
Basic needs	
Food	
Food on-site	158
Groceries	68
Extended cafeteria hours	1
Childcare	63
Pet care	1
Transportation	
Free parking	5
Oil change	1
Housing	7
Gift	
Toilet paper	2
Other (e.g., goody bags, coffee card)	4
Personal health and safe practice	
Mental health support	11
Scrubs	13
Shower area	2
Laundry bags for COVID-19 scrubs	1
Restroom for staff	1
PCR and antibody COVID-19 test	1
OTC medication available on site	2
Thermometers	1
Extended hours of occupational health	1
Financial support	
Extra money	4
Paid time off	15
All staff were in paid status	5
Assistance fund	3
Labor pool hours	1
Bereavement leave	1
Extended leave hours	1
Managerial support	
Extra/flexible staffing	2
Flexible schedule	4
Supervisors were more understanding with tardies/absences due to childcare	1
Communication	
Daily updates on PPE	1
Regular meetings for information sharing	1
Information on how to get COVID-19 test	1

*Note.* COVID-19 = coronavirus disease 2019; PCR = polymerase chain reaction; OTC medication = over-the-counter medication; PPE = personal protective equipment.

### Types of Resources Nursing Staff Reported Received From Hospitals During the COVID-19 Pandemic

Five major categories or types of resources emerged from participants who reported their hospitals provided additional resources during the pandemic (Table 1). A total of 382 statements regarding the types of resources were provided. The types of resources aligned with common categories of organizational resources and support described in previous literature (French et al., 2002; Ripp et al., 2020). These five major types of resources were basic needs, personal health and safe practice, financial support, managerial support, and communication.

The basic needs category included the most frequently reported resources, specifically food and childcare. The basic needs resources were those that meet daily necessities of life. These resources were offered in different ways or formats. Food, for example, was offered as food on-site, groceries, and extended access to purchase food items (e.g., extended cafeteria hours). Childcare was offered in the form of credits/funding to use toward childcare services, on-site childcare, or listings or connections to community childcare organizations that were available. Some of the less frequently identified resources under this category were transportation/parking and housing.

The second category was related to promoting and maintaining personal health and safe practice during the pandemic. Participants reported several resources related to mental health support that included free counseling and meditation. Other resources were directly related to personal health, such as providing polymerase chain reaction and antibody COVID-19 tests and availability of over-the-counter medications on-site. There were some resources that promoted safety for nursing staff and their families. These resources included the provision or laundering of hospital scrubs and shower areas at work. One participant responded, “Thermometers for those directly taking care of COVID patients and scrubs to change into when working directly with COVID patients.” Also, another participant reported the following: “We received a laundry bag for our COVID scrubs to securely take them home.”

The third category of resources represented financial support. These resources provide financial assistance beyond what was typically offered. The most frequently listed financial resource was paid time off. Less frequently mentioned resources included extra money or assistance funds, and different forms of extra hours or leave hours.

Participants also identified managerial support as a resource type, which is considered less tangible. Participants described receiving support and flexibility from their direct managers and particularly related to scheduling during the pandemic. One participant responded the following: “I’m lucky that my manager is pretty flexible when it comes to needing specific days off or letting me go home for family emergencies, which has thankfully been a very minimal amount.” Another participant said that “supervisors were more understanding with tardies/absences due to childcare with COVID.”

Somewhat related to managerial support, the fifth category represented communication. A small number of nurses reported their experiences related to daily updates on PPE, regular informational meetings with hospital and department leadership, and information on COVID-19 testing. Specific responses related to communication were the following: “twice weekly informational question/answer skype meetings with manager, department MD, hospital CNO” and “the hospital sent emails/ideas out for childcare coverage during the pandemic and information on who to call or where to go if we have symptoms and need to be tested.”

### Additional Emergent Themes Based on Nursing Staff’s Perceptions of Resources

In addition to the five main resource categories identified, four additional themes emerged from the responses: community support, unequal benefits, decreased resources, and insufficient PPE.

#### Community support

Many participants described resources provided by their communities. The responses showed a strong sense of community support for nursing staff. The most frequently listed community support resource was food; however, a few also identified other types such as childcare services. Participants were generally very explicit in noting the resources were from the community as opposed to their hospitals. For example, one participant reported, “The community did [provide resources]. The workplace did not,” while another participant stated “Not offered by the hospital directly, offered by the community. YMCA offered childcare. Community continues to provide food.” In some instances, participants noted that the hospital facilitated provision of resources by the community, “Food was donated by individuals to the hospital, and the hospital decided which units got the free food.” A few responses indicated how the hospital acted as a barrier to staff receiving community support “The community sent us meals; the hospital tried to stop it.”

#### Unequal benefits

While listing different types of resources provided by their hospitals, many participants added comments indicating that the resources did not benefit all nursing staff equally. Participants identified childcare as a resource provided by the organization but added that this resource did not help them as they had no children. A participant commented, “They provided opportunities to obtain childcare (I don’t need it).” In some cases, participants indicated that there were conditions to receive certain resources and consequently they were restricted or unable to benefit from them. For example, “They offered help with childcare I was not eligible for, since my child was at home daycare.”

In addition to differences in childcare benefits, participants also noted differences in the provision or availability of resources between day and night shifts. Certain resources provided by the organization such as grocery services or on-site food were often only available during daytime hours. Night shift workers did not benefit from these resources as examples mentioned: “Night shift is often left out” and “Rarely have meals during night shift and the cafeteria is closed. Groceries available in the cafeteria during the day.”

There were some perceived differences in resources based on their role in the organization. Travel nurses commented that the provided resources by the organization were not available to them: “As a travel nurse, they did not extend things to us, as they did to their staffed nurses.” Others described that resources were only provided to providers/physicians in the organization. One participant said,

Providers have demanded scrubs be provided by the hospital during this time, they have been given this accommodation. But nurses and CNAs who work consistently at the bedside have not and the organization has indicated will not be provided this benefit during this time, due to availability. Same applies with wanting to get tested, several nursing staff have requested testing and have been denied due to “lack of symptom,” but the organization turned around and offered routine testing/surveillance of providers. Definite double standard.

#### Decreased resources

Participants described a decrease in resources since the start of COVID-19. In some cases, this was a suspension or removal of resources that had been previously provided and were no longer available. Participants described things being “taken away,” such as access to food at work, hours and pay, and recognition programs. For example, a participant responded that “ . . . Food options were minimized (very limited hours of operation for the cafeteria) and takeout could not be ordered. No meals were provided.” Others, commenting on changes in work hours and pay during the pandemic, responded “ . . . they just took away our incentive pay, threatened our jobs and pay if we didn’t come to work.,” and “ . . . management has eliminated overtime and is cutting minutes anywhere they can.” Multiple participants mentioned the impact of COVID-19 on Nurses’ Week as follows: “they instead cancelled everything for nurse’s week” and “Nurses week was cancelled so no celebrations for all the nurses.”

While some resources were suspended or removed, participants also noted changes in resources as the pandemic progressed. Comments indicated that some resources had been provided initially but had decreased or were suspended as the pandemic continued. Some comments were as follows: “Food during working hours, snacks as we were leaving work most days. This has decreased since the beginning of COVID-19” and “We were receiving donations of food but then the hospital stopped allowing that.” These comments illustrate nursing staff perceptions that hospital support decreased or waned with time.

#### Insufficient PPE

The final theme that emerged from responses was a perception of insufficient PPE. Regardless of any type of resources provided, many participants commented that they were experiencing insufficient supplies of PPE during the pandemic. Quotes to illustrate this theme were as follows: “Still only getting one N95 and 1 surgical mask a week. Only allowed to wear N95 if doing procedure, not suspected or positive COVID for routine care,” and “Yes [we received resources]. my hospital has been wonderful and trying hard to protect us but also low on PPE.,” and “No [we did not receive any resources]. We did not even have the PPE we needed for the longest time.” These comments show how support must first address basic requirements of work (sufficient PPE) and then attention should be given to other resources to help alleviate the increased workload and burden during the pandemic.

## Discussion

This study was conducted to examine nursing staff’s perceptions of resources provided by hospitals during the COVID-19 pandemic and draw attention to ongoing organizational support needs to safeguard the health and well-being of nursing staff. We found that almost 42% of the sample did not report receiving any additional resources during the pandemic. Among nursing staff who received support, resources addressed basic needs, personal health and safe practice, financial and managerial support, and communication. Participants most frequently reported the basic needs resources that addressed availability of food on-site, groceries, and childcare support. These types of resources are congruent with previous studies (French et al., 2002; Ripp et al., 2020) and are considered critical to allay the fear and anxiety of nursing staff during the pandemic (Ripp et al., 2020; Shanafelt et al., 2020).

In our study, 211 out of 360 participants were provided with some type of organizational resources. Some participants made explicit responses differentiating between hospital support and community support. Our findings highlight the caring and supportive role of local communities toward nursing staff during the pandemic. Such statements also show perceptions of lack of support from hospitals. Earlier studies have shown when employees perceive the institution as not caring, supportive, and show concern toward their health and well-being, they were less likely to commit to their institution (Arshadi & Hayavi, 2013; Kurtessis et al., 2017). Nursing leaders are urged to allocate critical resources for nursing staff, where research shows that decreased employee commitment can lead to a high turnover and increase organizational cost (Fernet et al., 2017).

Our study found that many participants perceived the available resources did not benefit nursing staff equally. This finding can guide nursing leaders during planning and allocation of hospital resources. It supports moving beyond generic resources and tailoring them to staff needs where differences do exist by shift type (day vs. night) and dependent care (children vs. elderly) among others. There is strong scientific evidence that night shifts have detrimental effects on personal health and safety. Night shift nurses experience sleep loss, sleepiness, fatigue, and performance decrements and are at high risk for sickness absences, adverse health outcomes, and drowsy driving incidents (Akerstedt & Wright, 2009; Dall’Ora et al., 2020; Geiger-Brown et al., 2012; Scott et al., 2007; Vetter et al., 2016). The night shifts often place substantial burden on family relationships, childcare arrangements, and social life (Vitale et al., 2015). Organizational resources may be available to support nursing staff but may require some flexibility and tailoring to shift types. For example, nursing leaders may offer elderly care support for staff who do not have children as an alternative for childcare support. Besides providing basic needs such as food on-site, alternative resources that address the physiological challenges of night shifts can be made available to ensure more equitable provision of support resources. Nurse managers can allocate a private space for brief naps during the early morning hours, encourage more frequent rest breaks, provide coffee on-site, and explore the possibility of transportation services post night shifts.

During the COVID-19 pandemic, international studies have reported high psychological distress such as anxiety and depression, and post-traumatic stress among health care workers including nurses. Front-line health care staff who cared for patients with COVID-19 were more likely than others to experience decline in psychological well-being (Giusti et al., 2020; Lu et al., 2020; Que et al., 2020). Health care providers involved in the direct care of patients with severe acute respiratory syndrome suffered from post-traumatic stress long after the outbreak was over (Maunder et al., 2006). Despite critical need for mental health services, only 11 participants (approximately 3% of the sample) reported a specific mental health resource in this study. This is concerning as mental health resources are identified as a priority area for promoting and maintaining the emotional well-being of health care workers during the pandemic (Ripp et al., 2020). The recent National Action Plan to Advance Patient Safety also highlights the importance of developing programs to address psychological safety and burnout in health care workers (National Steering Committee for Patient Safety, 2020). Given the potential long-term psychological effects of this pandemic, nursing leaders are well positioned to raise awareness, expand programming, and facilitate access to mental health services for staff and particularly for those who cared for patients with COVID-19.

There are some limitations in this study. Participants were recruited through convenience sampling, and our data showed more representation from South and Midwest regions that may limit the generalizability of the findings. It is also possible to have sampling bias where participants were recruited from social media outlets and many felt strongly one way or another to report their experiences. In addition, we did not ask the nursing staff directly about the specific benefits or resources that their hospitals provided. The data collection relied on the recall of the participants of what they knew to be offered. Thus, their answers may not reflect all of the benefits that were actually offered by their organization. We used data from a single-item of the questionnaire and could not further explore staff perceptions, attitudes, and beliefs that may have provided a better understanding of employee expectations and needs during the pandemic.

### Implications for Occupational Health Nursing Practice

Our findings can help hospitals identify shortcomings in resources and prioritize and secure different types of resources perceived as meaningful to nursing staff. For basic needs that consist largely of perishables, it is important to fairly distribute them among staff on day and night shifts and across different nursing units. For personal health and safe practice, these resources address psychological support-mental health services and personal health like scrubs, PPE, screening tests, and laundering services. Although hospitals have made improvements in PPE shortages as these data were collected and made screening tests more available, nursing leaders remain the liaison between nursing staff and hospital administration and are responsible for facilitating access to COVID-19 screening tests and providing PPE. These resources are perceived as a priority and address worries related to safety and well-being. Critically, nursing staff are not reporting increased access to mental health resources, which is likely essential to maintaining long-term well-being and sustainability of the nursing workforce during and after this pandemic. Occupational health leaders should advocate for and implement programming to support the mental health of all nursing staff. Managerial support and communication are interrelated resources where nursing leaders can initiate unit-level debriefing sessions to address staff concerns about personal safety and health risks, and perceptions about available resources. These sessions may be conducted online to accommodate participation from all shifts and can be used to explore employee expectations and possible resources that are more meaningful to them such as childcare, dependent care, transportation, scrubs, and flexibility in scheduling.

## Conclusion

Our study provides important insights about nursing staff perceptions of hospital resources during the pandemic. We found that almost half of the participants did not report receiving any additional support. We quantified five major types of resources nursing staff received from hospitals among which basic needs such as availability of food on-site, groceries, and childcare were most frequently cited. Resources related to mental health services support were less mentioned particularly when international prevalence data demonstrates the psychological burden of the pandemic on nurses. Four themes emerged from the data related to nursing staff perceptions of organizational support: community support, unequal benefits, decreased resources, and insufficient PPE. The lessons from this study can assist in the planning and decision-making phase of hospital management and occupational health professionals to ensure sustainability and optimal performance of nursing staff during major crises.

**Applying Research to Occupational Health Practice**

This study provides an understanding of nursing staff perceptions of organizational resources provided by hospitals during the COVID-19 pandemic. Our findings call for attention to organizations to provide ongoing support needed to safeguard the health and well-being of hospital nursing staff. Occupational health practitioners should identify shortcomings in resources, and prioritize and secure different types of resources that are perceived as meaningful to nursing staff, particularly those that support mental health.
